# Thyroid dysfunction and dyslipidemia in chronic kidney disease patients

**DOI:** 10.1186/s12902-015-0063-9

**Published:** 2015-10-29

**Authors:** Saroj Khatiwada, Rajendra KC, Sharad Gautam, Madhab Lamsal, Nirmal Baral

**Affiliations:** Department of Pharmacy, Central Institute of Science and Technology (CIST) College, Pokhara University, Kathmandu, 44600 Nepal; Department of Biochemistry, B P Koirala Institute of Health Sciences, Dharan, Nepal

**Keywords:** Chronic kidney disease, Dyslipidemia, Nepal, Subclinical hypothyroidism, Thyroid dysfunction

## Abstract

**Background:**

Chronic kidney disease (CKD) is becoming a serious health problem; the number of people with impaired renal function is rapidly rising. Progression of CKD is associated with having a number of complications, including thyroid dysfunction, dyslipidemia and cardiovascular diseases. This study was conducted to investigate thyroid function and lipid profile in CKD patients.

**Methods:**

A cross-sectional study was conducted among 360 chronic kidney disease patients at B P Koirala Institute of Health Sciences, Dharan, Nepal. Demographic features (age and sex) and medical history of diabetes mellitus, hypertension and cardiovascular diseases of each patient were noted, and blood samples (5 ml) were analyzed for serum urea, creatinine, glucose, free triiodothyronine (T3), free thyroxine (T4), thyroid stimulating hormone (TSH), total cholesterol, high density lipoprotein (HDL) cholesterol, low density lipoprotein (LDL) cholesterol and triglyceride.

**Results:**

Thyroid dysfunction was found in 38.6 % CKD patients, the most common being subclinical hypothyroidism (27.2 %), followed by overt hypothyroidism (8.1 %) and subclinical hyperthyroidism (3.3 %). Hypercholesterolemia, low HDL cholesterol, undesirable LDL cholesterol and hypertriglyceridemia were observed in 34.4, 34.1, 35 and 36.6 % patients respectively. Stage 4 and 5 CKD patients had significantly higher risk of having thyroid dysfunction as compared to stage 3 patients. Significant risk factors for cardiovascular disease in CKD patients included presence of diabetes mellitus, hypercholesterolemia, undesirable LDL cholesterol and being in stage 4 and 5 (as compared to stage 3).

**Conclusions:**

Thyroid dysfunction, hypercholesterolemia, low HDL cholesterol, undesirable LDL cholesterol and hypertriglyceridemia are common in CKD patients. Progression of CKD is accompanied by rise in hypothyroidism and cardiovascular disease.

## Background

Chronic kidney disease (CKD) is becoming a serious health problem; the number of people with impaired renal function is rapidly rising, especially in industrialized countries [1]. Recent reports, however, suggest an abrupt rise in CKD in developing countries from Asia due to increase in concomitant diseases such as type 2 diabetes, hypertension and cardiovascular diseases (CVDs) [2]. Associated with rise in CKD numbers is the extensive increase in the health cost for management of CKD especially of 5^th^ stage [3].

Reports suggest that progression of CKD is associated with having a number of complications, including thyroid dysfunction, dyslipidemia and CVD [4]. The kidney normally plays an important role in the metabolism, degradation and excretion of thyroid hormones. CKD affects the hypothalamus pituitary thyroid axis. CKD affects thyroid function in many ways, including low circulating thyroid hormone levels, altered peripheral hormone metabolism, insufficient binding to carrier proteins, reduced tissue thyroid hormone content and altered iodine storage in the thyroid gland. Thus, in CKD, thyroid hormone metabolism is impaired [5]. CKD is associated with a higher prevalence of primary hypothyroidism, both overt and subclinical, but not with hyperthyroidism. Prevalence of primary hypothyroidism, mainly in the subclinical form, increases as glomerular filtration rate (GFR) decreases [6].

Dyslipidemia has been established as a well-known traditional risk factor for CVD in CKD and large-scale observational studies have shown that total and low-density lipoprotein (LDL) cholesterol are the most important independent predictors of cardiovascular morbidity and mortality [7]. Several factors contribute to the development dyslipidemia associated with chronic renal impairment. Patients with CKD have a reduction in the activity of lipoprotein lipase and hepatic triglyceride lipase. This interferes with uptake of triglyceride rich, apolipoprotein B containing lipoproteins by the liver and in peripheral tissue, yielding increased circulation of these atherogenic lipoproteins [4]. Progression of CKD is accompanied by the development of specific alterations of the lipoprotein metabolism [8]. Reports show that mortality due to CVD was 10–30 times higher in dialysis patients than in the general population [9].

There is growing evidence that abnormalities in lipid metabolism may contribute to renal disease progression [10]. Thyroid dysfunction and dyslipidemia in CKD may further increase CVD risk leading to increased morbidity and mortality. Hence, early diagnosis of thyroid and lipid disorders by regular screening, and treatment of such disorders in CKD patients may be highly beneficial to slow the progression of CKD, in addition to the prevention of CVD risk [4, 6]. A study in eastern Nepal, reported CKD in 10.6 % of general community with age and diabetes mellitus being the significant predictors for CKD [11]. Another small study reported dyslipidemia to be very common in Nepalese CKD patients [12]. In view of the decreased thyroid function and dyslipidemia in CKD patients, and the lack of data for thyroid function and dyslipidemia in Nepalese population with CKD, we undertook the present study in the population with CKD.

## Methods

A cross-sectional study was conducted among CKD patients attending biochemistry laboratory of B P Koirala Institute of Health Sciences, Dharan, Nepal from February 2012 to March 2014 to investigate thyroid function and lipid profile in CKD stage 3–5. A total of 360 newly diagnosed and known CKD cases (stage 3 to stage 5) were included in the study. CKD was defined on the basis of National Kidney Foundation guidelines of having an estimated glomerular filtration rate (eGFR) < 60 ml/min/1.732 m^2^ for more than 3 months. The Modification of Diet in Renal Disease study (MDRD) equation was used to calculate eGFR [13]. Patients with known thyroid disorders, on medications affecting thyroid function and on lipid lowering agents were excluded from the study. The study protocol was approved by the Institutional Review Board of the B P Koirala Institute of Health Sciences, Dharan and consents were obtained from each patient.

Demographic features (age and sex) and medical history of diabetes mellitus, hypertension and CVD of each patient were noted, and blood samples (5 ml) were collected. CVD was defined as a history of coronary heart disease, congestive heart failure, peripheral arterial disease or stroke. Blood was analyzed for serum urea, creatinine, glucose, total cholesterol, high density lipoprotein (HDL) cholesterol, low density lipoprotein (LDL) cholesterol, triglyceride, triiodothyronine (T3), thyroxine (T4) and thyroid stimulating hormone (TSH). Serum urea level and blood glucose were estimated using enzymatic methods and creatinine by Jaffe method. Serum free T3, free T4 and TSH were measured by using fluorescent immunoassay (VIDAS, biomeriux SA, France). Similarly, total cholesterol, HDL cholesterol, LDL cholesterol and triglycerides were measured using kits from AGAPPE diagnostics by Biolyzer 100.

Thyroid dysfunction was considered if patients thyroid hormones fall outside the reference range; free T3 (4.0–8.3 pmol/L), free T4 (9.0–20.0 pmol/L) and TSH (0.25–5 mIU/L). Euthyroid was considered if thyroid hormone levels fall within reference range. Overt hypothyroidism was defined as TSH > 5 mIU/L and free T3 < 4.0 pmol/L and free T4 < 9.0 pmol/L. Subclinical hypothyroidism was considered if TSH > 5 mIU/L and free T3 and free T4 within reference range. Subclinical hyperthyroidism was defined as TSH < 0.25 mIU/L and free T3 and free T4 within reference range. The data generated from study was entered into MS excel and analyzed using SPSS version 11.0. The data were expressed as mean ± SD (for normal distribution) and median (for non-normal distribution) for continuous variables and as percentage (number) for categorical variables. One way ANOVA and Kruskal Wallis test were applied for continuous variables and chi square test for categorical variables at 95 % confidence interval. Odds ratio with 95 % confidence interval (CI) was calculated to assess different risk factors for thyroid dysfunction and CVD in CKD patients.

## Results

The study population comprised 360 CKD patients with 53.8 % males and 46.1 % females. The mean age of study population was 44.1 ± 16.4 years. Stage 3, stage 4 and stage 5 CKD patients were 40 % (*n* = 144), 43.3 % (*n* = 156) and 16.6 % (*n* = 60) respectively. Various clinical and biochemical parameters of study population according to CKD stages are shown in Table 1. Diabetes and CVD prevalence in CKD showed significantly increasing trend from stage 3 to 5. Blood urea, creatinine and TSH level increased significantly across CKD stages 3–5. Prevalence of diabetes mellitus, hypertension and CVD was; 45.8, 63.8 and 29.8 % in stage 3; 40.3, 72.4 and 51.9 % in stage 4; and 60, 80 and 75 % in stage 5 CKD patients respectively.

**Table 1:** Characteristics of study patients according to CKD stage

Variables	All patients *N* = 360	Stage 3 *N* = 144	Stage 4 *N* = 156	Stage 5 *N* = 60	*P* value
Age (Years)	44.1 ± 16.4	41.9 ± 16.1	47.5 ± 15.6	40.3 ± 17.7	0.002
Sex					
Male	53.8 % (194)	18.8 % (68)	25.2 % (91)	9.7 % (35)	0.117
Female	46.1 % (166)	21.1 % (76)	18.0 % (65)	6.9 % (25)	
Diabetes mellitus	45.8 % (165)	18.3 % (66)	17.5 % (63)	10 % (36)	0.035
Hypertension	70.2 % (253)	25.5 % (92)	31.3 % (113)	13.3 % (48)	0.053
Cardiovascular disease	46.9 % (169)	11.9 % (43)	22.5 % (81)	12.5 % (45)	<0.001
Urea (mg/dL)	106.3 ± 60.1	71.3 ± 39.5	100.7 ± 37.7	204.7 ± 39.5	<0.001
Creatinine (mg/dL)	4.0 ± 3.4	1.8 ± 0.3	3.3 ± 0.5	10.9 ± 3.2	<0.001
eGFR (ml/min per 1.73 m^2^)	28.2 ± 15.3	44.0 ± 8.9	22.0 ± 4.0	6.4 ± 2.6	<0.001
Free T3 (pmol/L)	4.9 ± 1.1	5.0 ± 1.1	4.9 ± 1.1	4.7 ± 0.8	0.131
Free T4 (pmol/L)	11.6 ± 3.2	11.9 ± 3.2	11.5 ± 3.3	11.0 ± 2.6	0.175
TSH (mIU/L)	3.2 (1.8, 6.8)	2.5 (1.6, 4.3)	3.3 (1.7, 7.3)	5.4 (2.3, 8.8)	<0.001
Glucose (mg/dL)	117.4 ± 44.6	119.9 ± 45.6	116.7 ± 46.7	113.0 ± 36.1	0.596
Total cholesterol (mg/dL)	191.9 ± 31.7	194.2 ± 32.9	189.9 ± 31.3	191.6 ± 29.4	0.501
HDL cholesterol (mg/dL)	42.1 ± 5.9	42.0 ± 5.8	42.0 ± 6.0	42.7 ± 6.1	0.718
LDL cholesterol (mg/dL)	103.6 ± 28.1	106.1 ± 30.9	100.6 ± 25.6	105.0 ± 26.7	0.215
Triglyceride (mg/dL)	205.7 ± 73.9	205.9 ± 79.8	207.1 ± 70.4	201.6 ± 68.8	0.884

The data is presented as mean ± SD (except for TSH level which showed non-normal distribution and is expressed as median) for continuous variables and as percentage (number) for categorical variables. *P* value was calculated at 95 % confidence interval among CKD stages 3–5 for different variables using one way ANOVA and Kruskal Wallis test for continuous variables, and chi square test for categorical variables

Thyroid function status and dyslipidemia according to CKD stages is shown in Table 2. Thyroid dysfunction was found in 38.6 % CKD patients, the most common thyroid dysfunction being subclinical hypothyroidism (27.2 %). Subclinical hypothyroidism got significantly common with CKD progression. Prevalence of subclinical hypothyroidism in stage 3, stage 4 and stage 5 was 15.2, 32.0 and 43.3 % respectively. Hypercholesterolemia (total cholesterol > 200 mg/dL), low HDL (HDL cholesterol < 40 mg/dL), undesirable LDL (LDL cholesterol > 100 mg/dL) and hypertriglyceridemia (triglyceride > 200 mg/dL) was; 38.8, 33.3, 38.8 and 34.7 % in stage 3; 32.6, 35.8, 32.0 and 38.4 % in stage 4; and 28.3, 31.6, 33.3 and 36.6 % in stage 5 respectively.

**Table 2:** Thyroid dysfunction and dyslipidemia in different CKD stages

Variables	All patients *N* = 360	Stage 3 *N* = 144	Stage 4 *N* = 156	Stage 5 *N* = 60	*P* value
Thyroid status					
Euthyroidism	61.4 % (221)	29.4 % (106)	24.7 % (89)	7.2 % (26)	<0.001
Subclinical hypothyroidism	27.2 % (98)	6.1 % (22)	13.9 % (50)	7.2 % (26)	<0.001
Overt hypothyroidism	8.1 % (29)	2.5 % (9)	3.3 % (12)	2.2 % (8)	0.232
Subclinical hyperthyroidism	3.3 % (12)	1.9 % (7)	1.4 % (5)	-	0.21
Hypercholesterolemia (Total cholesterol > 200 mg/dL)	34.4 % (124)	15.5 % (56)	14.1 % (51)	4.7 % (17)	0.292
Low HDL cholesterol (HDL cholesterol <40 mg/dL)	34.1 % (123)	13.3 % (48)	15.5 % (56)	5.2 % (19)	0.811
Undesirable LDL cholesterol (LDL cholesterol > 100 mg/dL)	35 % (126)	15.5 % (56)	13.8 % (50)	5.5 % (20)	0.443
Hypertriglyceridemia (Triglyceride > 200 mg/dL)	36.6 % (132)	13.8 % (50)	16.6 % (60)	6.1 % (22)	0.798

The data is presented as percentage (number). *P* value was calculated among CKD stages 3–5 for different variables using chi square test at 95 % confidence interval

The odds ratio of having hypercholesterolemia, low HDL, undesirable LDL and hypertriglyceridemia in stage 4 patient was 0.76 (95 % CI: 0.47–1.22, *p* = 0.263), 1.12 (95 % CI: 0.69–1.8, *p* = 0.641), 0.74 (95 % CI: 0.46–1.19, *p* = 0.216) and 1.17 (95 % CI: 0.73–1.88. *p* = 0.502) respectively as compared to stage 3 patients. Similarly, the odds ratio of having hypercholesterolemia, low HDL, undesirable LDL and hypertriglyceridemia in stage 5 patient was 0.62 (95 % CI: 0.32–1.19, *p* = 0.152), 0.92 (95 % CI: 0.48–1.76, *p* = 0.817), 0.78 (95 % CI: 0.41–1.47, *p* = 0.455) and 1.08 (95 % CI: 0.58–2.03, *p* = 0.791) respectively as compared to stage 3 patients.

The odds ratio of having thyroid dysfunction and CVD in CKD patients due to various risk factors is shown in Table 3. There were significant odds of having thyroid dysfunction in stage 4 and 5 as compared to stage 3 CKD. Presence of diabetes, hypercholesterolemia and undesirable LDL cholesterol had significant odds of having CVD. Stage 4 and 5 patients had significant odds of having CVD as compared to stage 3 patients. The odds of having CVD in CKD patients with thyroid dysfunction was 1.25 (95 % CI: 0.81–1.91, *p* = 0.303) as compared to euthyroids. eGFR had significant positive correlation with free T3 (*r* = 0.11, *p* = 0.036), free T4 (*r* = 0.119, *p* = 0.024) and significant negative correlation with TSH (*r* = −2.01, *p* < 0.001) but no correlation with parameters of lipid profile.

**Table 3:** Risk factors for thyroid dysfunction and cardiovascular disease in CKD

	Risk factors	Odds ratio (95 % CI)	*P* value
Thyroid dysfunction	Female gender	1.26 (0.82–1.92)	0.287
	Age > 60 years	1.53 (0.92–2.55)	0.098
	Diabetes mellitus	0.96 (0.63–1.48)	0.878
	CKD stage		
	Stage 3	1 (reference)	
	Stage 4	2.1 (1.28–3.42)	0.003
	Stage 5	3.64 (1.94–6.85)	<0.001
Cardiovascular disease	Hypertension	1.25 (0.79–1.97)	0.328
	Diabetes mellitus	1.93 (1.26–2.94)	0.002
	Hypercholesterolemia	4.95 (3.08–7.97)	<0.001
	Low HDL cholesterol	1.06 (0.68–1.64)	0.779
	Undesirable LDL cholesterol	2.83 (1.8–4.43)	<0.001
	CKD stage		
	Stage 3	1 (reference)	
	Stage 4	2.53 (1.57–4.08)	<0.001
	Stage 5	7.04 (3.55–13.97)	<0.001

Hypercholesterolemia, low HDL and undesirable LDL were considered if total cholesterol > 200 mg/dL, HDL cholesterol < 40 mg/dL and LDL cholesterol > 100 mg/dL respectively. Stage 3 CKD was considered as reference for calculating odds for thyroid dysfunction and CVD in stage 4 and stage 5 CKD

## Discussion

The present study identifies thyroid dysfunction and dyslipidemia as a common disorder in Nepalese CKD patients. Thyroid dysfunction was found in 38.6 % CKD patients, the most common being subclinical hypothyroidism (27.2 %), followed by overt hypothyroidism (8.1 %) and subclinical hyperthyroidism (3.3 %). We found higher prevalence of thyroid dysfunction in CKD patients than shown by other studies. A small study in hemodialysis patients in western Nepal showed the combined prevalence of subclinical and clinical hypothyroidism in 26.6 % patients [14]. Study by Lo et al. found that the prevalence of hypothyroidism increased with lower levels of GFR (in units of mL/min/1.73 m^2^), occurring in 5.4 % of subjects with GFR greater than or equal to 90, 10.9 % with GFR 60–89, 20.4 % with GFR 45–59, 23.0 % with GFR 30–44, and 23.1 % with GFR < 30 (*p* < 0.001 for trend). They reported that 56 % of hypothyroidism cases were subclinical [6]. In a study in India among end stage renal failure (ESRD) patients, prevalence of subclinical hypothyroidism was 24.8 % [15]. A study by Ng et al. in peritoneal dialysis (PD) patients of Taiwan reported that, 98 (80.3 %) were having euthyroidism; 19 (15.6 %), subclinical hypothyroidism; and 5 (4.1 %), subclinical hyperthyroidism [16]. High rate of thyroid dysfunction in CKD patients as observed in our study may also be due to high prevalence of thyroid autoimmunity in study population, excess iodine nutrition or iodine deficiency, and the inclusion of subjects with non thyroidal illness [17, 18].

We observed decreasing trend for free T3 and free T4 levels (though the decrease were not significant), and increasing trend for TSH level (significant rise) across CKD stages 3–5, which suggest that TSH level increases with the progression of renal impairment (which is indicated by a decrease in GFR). Prevalence of subclinical hypothyroidism was much common in stage 5 (43.3 %), than stage 4 (32.0 %) and stage 3 (15.2 %) patients, which clearly demonstrates the parallel rise in hypothyroidism with CKD progression. Our findings, are similar to the study by Song et al. who found that there was an increasing trend for the population of low T3 according to the increase of a CKD stage (eGFR ≥ 90, 8.2 %; ≥60 eGFR < 90, 10.9 %; ≥30 eGFR < 60, 20.8 %; ≥15eGFR < 30, 60.6 %; eGFR < 15, 78.6 %) [19]. In the present study, we found that stage 4 and 5 patients had significantly high risk for thyroid dysfunction as compared to stage 3 patients, which is consistent with the findings of Lo et al., who observed increased risk for hypothyroidism with the decrease in GFR [6]. Song et al., also observed a positive relationship between eGFR and serum T3 and after adjusting for age and sex, compared with eGFR ≥ 60 ml/min/1.73 m^2^, eGFR < 60 ml/min/1.73 m^2^ was associated with an increased odds of low T3 (odds ratio 2.40) [19]. In a study in Saudi Arabia, there was a significant decrease in the levels of serum total T3, total T4 and total protein and albumin levels in CKD patients when compared with the controls. There was a significant increase in the level of TSH in the CKD patients compared with the controls [20]. However, a study in undialyzed CKD patients found that both T3 and T4 were significantly reduced whereas TSH remains to be unchanged in patient group compared to controls [21].

We found subclinical hypothyroidism (27.2 %) to be very common in CKD patients. It has been estimated that the prevalence of subclinical hypothyroidism ranges between 4 and 10 % in the general population and it has been well observed that hypothyroidism (overt or clinical) increases the risk for CVD. Subclinical hypothyroidism has been identified as a strong predictor of all-cause mortality in chronic dialysis patients and as a risk factor for nephropathy and cardiovascular events in type 2 diabetic patients [22]. In the present study, however, we did not observed significant risk of having CVD in CKD patients with thyroid dysfunction especially subclinical hypothyroidism as compared to those with normal thyroid function. A number of studies have reported that subclinical hypothyroidism is associated with an increased risk of coronary heart disease and there appears to be a significant increase in a cluster of metabolic CVD risk factors among people with subclinical hypothyroidism [23, 24]. The reduced T3 level, the reduced free T4 levels along with an elevated TSH raises the possibility of benefit from thyroid supplementation in CKD. However, it is still debatable about the importance and necessity of thyroid hormone supplementation in CKD patients and concrete evidence is not available for recommending supplementation of thyroid hormones in CKD patients [25].

In general, the prevalence of hyperlipidemia increases as renal function declines, with the degree of hypertriglyceridemia and elevation of LDL cholesterol being proportional to the severity of renal impairment [4]. CKD affects lipoprotein metabolism, leading to hypercholesterolemia, hypertriglyceridemia and excess LDL cholesterol [26]. In the present study, we reported dyslipidemia in significant number of CKD patients. Hypercholesterolemia, low HDL, undesirable LDL and hypertriglyceridemia was observed in 34.4, 34.1, 35 and 36.6 % patients respectively. Our finding are similar to the results of a study in Kathmandu, Nepal, where hypercholesterolemia, low HDL cholesterol, high LDL cholesterol and hypertriglyceridemia was present in 33.75, 32.5, 38.03 and 35.58 % CKD patients respectively, and CKD patients had higher odds of developing dyslipidemia as compared to non-CKD controls [12]. We however, did not found that stage 4 and stage 5 patients have significant odds of developing dyslipidemia as compared to stage 3 patients. Many studies have reported rise in level of lipid parameters and dyslipidemia prevalence in CKD patients, which may further assist in renal disease progression [10]. Study by Raju et al. found that there was a significant increase of serum triglycerides and very low density lipoprotein (VLDL) with a decrease in serum HDL cholesterol in both non dialysis and hemodialysis groups of CKD patients when compared with control. But there was no alteration in serum total cholesterol and LDL cholesterol in both groups [27].

Dyslipidemia in CKD may have significant impact, especially for CVD risk [7]. Our finding suggest for the significant association between the presence of cardiovascular disease with hypercholesterolemia and high LDL cholesterol in CKD patients. Our study reveals that CKD progression is strongly associated with CVD prevalence. Stage 4 and stage 5 patients had higher risk for having CVD as compared to stage 3 patients. The study however, did not find significant rise in dyslipidemia cases during CKD progression. A large number of epidemiologic studies have suggested the independent role of dyslipidemia on cardiovascular morbidity and mortality in the general population [10]. But, the role of dyslipidemia in the pathophysiology of atherosclerotic disease in patients with CKD remains controversial. Some studies have shown a positive association between cholesterol values and the risk for cardiovascular events in CKD individuals, whereas others failed to find any significant correlation. Although the precise causes of this significant deviation from what is observed in the general population have not been established, it has been proposed that the presence of phenomena such as inflammation or protein energy wasting (conditions very common in ESRD patients) may significantly confound the relationship between the traditional risk factors for CVD and mortality in CKD patient population [26]. Study by Chen et al. demonstrated that certain levels of dyslipidemia were independently associated with renal replacement therapy and rapid renal progression in CKD stage 3–5. Thus, assessment of lipid profile in CKD patients may help identify high risk groups with adverse renal outcomes [10]. Among, the various risk factors for CVD we considered, only diabetes had significant association with CVD occurrence. Diabetic condition had also significant association with progression of CKD. Diabetes is said to be the leading cause of CKD in many populations and is associated with excessive cardiovascular morbidity and mortality [28].

Thyroid disorders and CKD are independently some of the most prominent medical conditions found in patients in many countries. Clinicians, including nephrologists, must consider the dangers of thyroid disease and its appropriate treatment in conjunction to treating CKD. Thyroid dysfunction causes significant changes in kidney function and kidney diseases can be associated with thyroid disorders. Thus, both CKD and thyroid dysfunction mutually influence each other [29]. Similarly, dyslipidemia is the leading risk factor for CVD in CKD patients, and CVD remains the leading cause of death in CKD patients. Kidney Disease Outcomes Quality Initiative (K/DOQI) working group has suggested that, all adults and adolescents with CKD should be evaluated for dyslipidemias because of high risk for CVD [30].

Our findings of present study have great clinical significance. It suggest for the importance of regular screening and treatment of thyroid dysfunction and dyslipidemia in patients with CKD, which may further help to prevent CVD risk. This would help in better clinical management of CKD patients and thus better quality of life. So, we recommend for regular checkup of thyroid function and lipid profile in CKD patients. It should be however noted that, since the study was conducted in a region of Nepal where conditions (nutrition status, disease status, thyroid autoimmunity status, genetic components) of patients may be slightly different than other parts of world, the present findings may vary somewhat for other countries. The present study has some limitations which should be noted. First, patients with non thyroidal illness (which is typically seen in some ill patients, including those with end-stage renal disease) were not identified, since thyroid testing was done only once. This may have resulted in higher rate of thyroid dysfunction in the present study due to inclusion of some patients with non thyroidal illness. Second, the iodine status of the subjects was not studied, so excess iodine nutrition or iodine deficiency in these regions may contribute to thyroid disorders, mainly subclinical hypothyroidism [18]. Third, we did not assess thyroid autoimmunity status in the study population. High prevalence of thyroidal autoimmunity is related with increased thyroid dysfunction, so high rate of thyroid autoimmunity (if present) may have contributed for high thyroid dysfunction rate in the present study [17]. Further studies should explore potential causal mechanisms through which CKD may be associated with increased TSH and reduced thyroid function, including the possible roles of autoimmunity and iodine excess, and the mechanism of dyslipidemia in patients with CKD.

## Conclusions

In summary, the present study finds thyroid dysfunction to be very common in CKD patients and reveals the significant association between CKD progression and thyroid dysfunction. The study also finds dyslipidemia as a common disorder in CKD. The study reveals that CKD patients with elevated lipid parameters have strong risk for developing CVD, so early treatment for lipid abnormalities in CKD patients may lower the chance of developing CVD later.

## Abbreviations

CHD: coronary heart disease
CKD: chronic kidney disease
CVD: cardiovascular disease
eGFR: estimated glomerular filtration rate
ESRD: end stage renal disease
FT3: free triiodothyronine
FT4: free thyroxine
GFR: glomerular filtration rate
HDL: high density lipoprotein
LDL: low density lipoprotein
MDRD: modification of diet in renal disease study
TSH: thyroid stimulating hormone
